# Customized Vestibular Rehabilitation for Vestibular Schwannoma Excision via Translabyrinthine Approach: A Single-Center Experience

**DOI:** 10.3390/jcm13144183

**Published:** 2024-07-17

**Authors:** Virginia Fancello, Elisabetta Rebecchi, Anna Lisa Giannuzzi, Giuseppe Fancello, Simone Faroldi, Luca Rosani, Mario Sanna

**Affiliations:** 1 Gruppo Otologico, Otology and Skull Base Surgery, Via Morigi, 41, 29121 Piacenza, Italy; 2 ENT Department, University Hospital of Sassari—AOU SS, 07100 Sassari, Italy

**Keywords:** vestibular rehabilitation, vestibular schwannoma, video head impulse test, functional head impulse test

## Abstract

**Objectives:** To evaluate the effectiveness of intensive customized vestibular rehabilitation after vestibular schwannoma (VS) excision. **Methods:** 52 patients who underwent VS removal via a translabyrinthine approach from 2020 to 2022 were involved in this study. Bedside examination, video head impulse test (vHIT), functional head impulse test (fHIT), and the dizziness handicap inventory (DHI) were performed before and after the rehabilitation, which consisted of 10 sessions of specifically designed vestibular, visual, and physical integrated training. **Results:** After rehabilitation, the vHIT showed overall unchanged values on the affected and healthy side. In contrast, the scores of fHIT, which explores the higher connection of the vestibular system with visual and cerebellar pathways, improved on both the pathological and healthy sides after training (*p*-value 0.004 and 0.000, respectively). The effectiveness of the rehabilitation was reinforced by the DHI scores, which were considerably lower after training. **Conclusions:** To our knowledge, this is the first study to explore fHIT outcomes after removal of VS, estimating the impact of rehabilitation on the overall compensation process. The outcomes support the role of extensive postsurgical rehabilitation in the compensatory process, even just a few days after surgery.

## 1. Introduction

Vestibular schwannoma (VS), also called acoustic neuroma, is a benign Schwann cell-derived tumor arising, in 85% of cases, from the inferior vestibular nerve [1].

VS, despite being benign, poses a risk to several intracranial structures because of the mass effect. The natural history of tumor growth impacts the VIII and VII cranial nerves, inner ear, and brainstem.

Over 60% of patients report having tinnitus and gradual hearing loss as their main symptoms. Larger tumors have the potential to produce hydrocephalus and brainstem compression.

Various approaches exist to manage patients with VS, such as ‘watch and rescan’, surgical removal, and radiotherapy. 

The goal of the surgical treatment is to remove the tumor and thus prevent mass effect [2].

Despite considerable progress in diagnostics and therapy, the natural history of the tumor and the surgery might have a significant impact on quality of life. One of the major issues is the resulting unilateral vestibular deafferentation, which may cause vertigo, dizziness, and impaired balance [3].

The decision on surgical excision in our hospital performed via translabyrinthine approach, is based on tumor size, growth, and clinical manifestations. 

Acute vestibular dysfunction is a major issue that often characterizes the clinical course in the peri- and post-operative periods. After the VS is surgically removed, vestibular function decreases dramatically. Most patients experience vertigo and impaired postural control in the days immediately after surgery. 

Following the surgical procedure, patients are initially admitted to a sub-intensive care unit before being transferred to a ward as soon as their general health stabilizes.

Long periods of inactivity and immobility are the primary causes of muscular deconditioning, as well as feelings of exhaustion, depression, and difficulty recovering. 

In addition, the loss of vestibular function is known not only to affect the vestibulo-ocular and vestibulo-spinal reflexes but also cognition and spatial information processing [4]. Vestibular deafferentation is postulated to have long-term effects on hippocampal, insular, and parietal connections, thus favoring a decline in cognitive function [5].

To avoid extended bed rest and subsequent complications, we begin mobilization and rehabilitation immediately after VS excision. Early patient mobilization is well established to shorten hospital stays, speed up recovery, decrease the risk of complications following surgery, and accelerate the return to functional independence, decreasing long-term healthcare costs [6].

To accomplish these goals, we took advantage of the facilities offered by our hospital, which include physical and vestibular therapy. Thus, we designed a specific vestibular rehabilitation program, which became a standard of care in subjects affected by dizziness and vertigo after the surgical procedure. 

A preliminary comprehensive evaluation of balance is crucial. 

The process of compensation after vestibular deafferentation typically takes up to several months to occur and can be slowed down by aging, neurologic, ophthalmologic, and osteo-muscular conditions.

Rehabilitation, which plays a crucial role, consists of postural exercises, vestibula-ocular reflex (VOR) training, visual training, working memory training, and a combination of thereof. 

Modern vestibular testing tools help detect latent vestibular lesions with accuracy, as well as determine the vestibular compensation state and obtain multiple information. 

The video head impulse test (vHIT) is used to measure semicircular canal function, which is computed as the ratio of eye movement reaction to a sudden head rotation, the key indicator of the VOR function [7].

The functional head impulse test (fHIT) analyses the multisensory integration of the VOR and its central multimodal processing, as a result of connections between the cerebral, vestibular, and visual systems. This test assesses the ability to identify the orientation of a Landolt C that quickly appears on a screen while the examiner applies passive head accelerations to the patient [8].

Both vHIT and fHIT are easy to perform and essential for clinical diagnosis, treatment, rehabilitation, and research; moreover, these tests provide a complementary and effective background for objective and precise assessment of vestibular function [9,10,11].

The purpose of this study is to assess the impact of our rehabilitation program in adult patients who had vestibular schwannoma excision utilizing subjective tools such as DHI and objective data obtained from vHIT and fHIT outcomes.

## 2. Materials and Methods 

We evaluated adult patients who underwent vestibular schwannoma excision via a translabyrinthine approach (TLA) from 2020 to 2022. 

**Figure 1:**
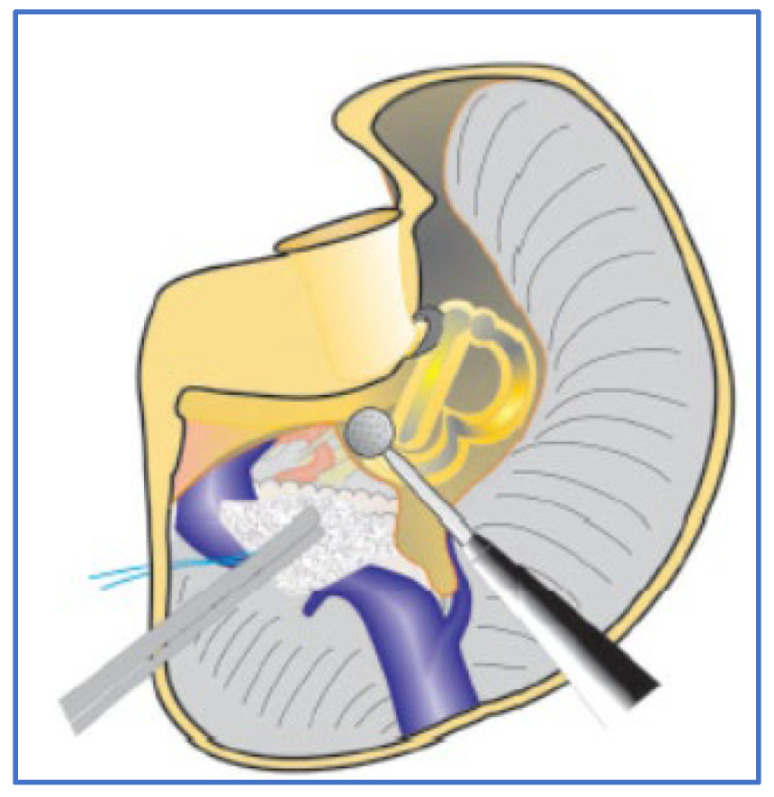
Enlarged TLA, including labyrinthectomy (left side); courtesy of Gruppo Otologico.

The surgical approach consisted of an enlarged TLA, which included mastoidectomy, labyrinthectomy, and identification of the facial nerve using the ampullary nerve as a landmark, lowering the high jugular bulb, and extending bone removal around and anterior to the internal acoustic canal, to dominate the surgical field (Figure 1). 

The labyrinthectomy involves drilling both the lateral and superior semicircular canals. However, the anterior part of the ampullae of these two canals is left intact to safeguard the facial nerve’s labyrinthine segment and to serve as a landmark for the superior vestibular nerve [12].

The exclusion criteria from this study were revision surgery, previous gamma knife treatment, residual VS, or the presence of a cochlear implant. The tumor size ranged from 1.5 to 3 cm; patients with VS larger than 3 cm were not included in this study, as well as those affected by neuromuscular or ophthalmic diseases.

In total, 52 patients were involved in this study. 

All patients provided informed consent for the surgical treatment and for the rehabilitation, which was started in a range of time that varies from 3 to 7 days after surgery. 

The dizziness handicap inventory (DHI) was used to assess the subjective degree of functional, emotional, and physical dizziness of each patient before and after their rehabilitation [13].

Each patient underwent a thorough vestibular assessment which included a bedside examination (BSE), vHIT, and fHIT. 

The customized rehabilitation with vestibular and visual tasks was performed by means of a BEON Solution System, under the supervision of experienced physiotherapists, and consisted of 10 sessions (one per day).

**Figure 2:**
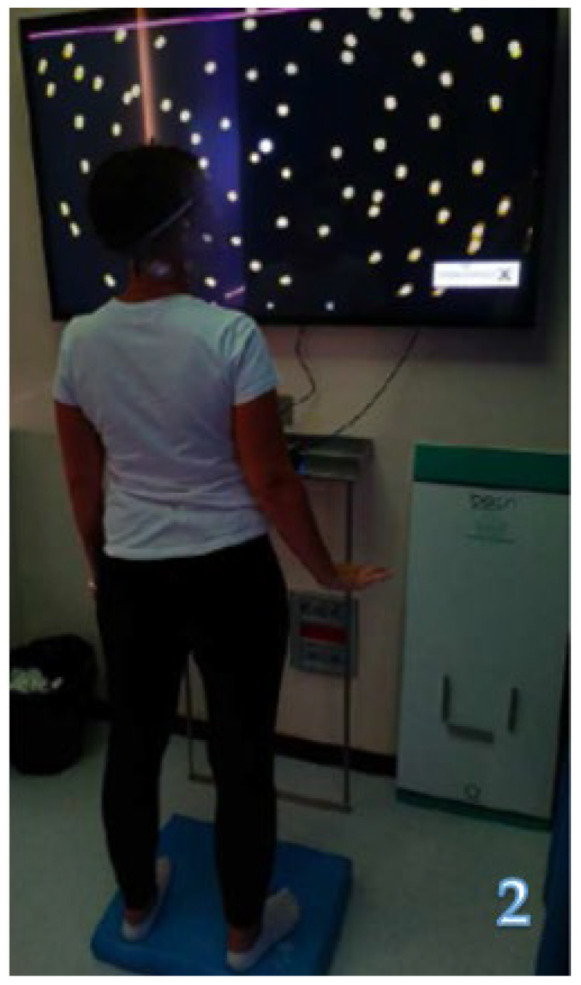
v-Gym.

**Figure 3:**
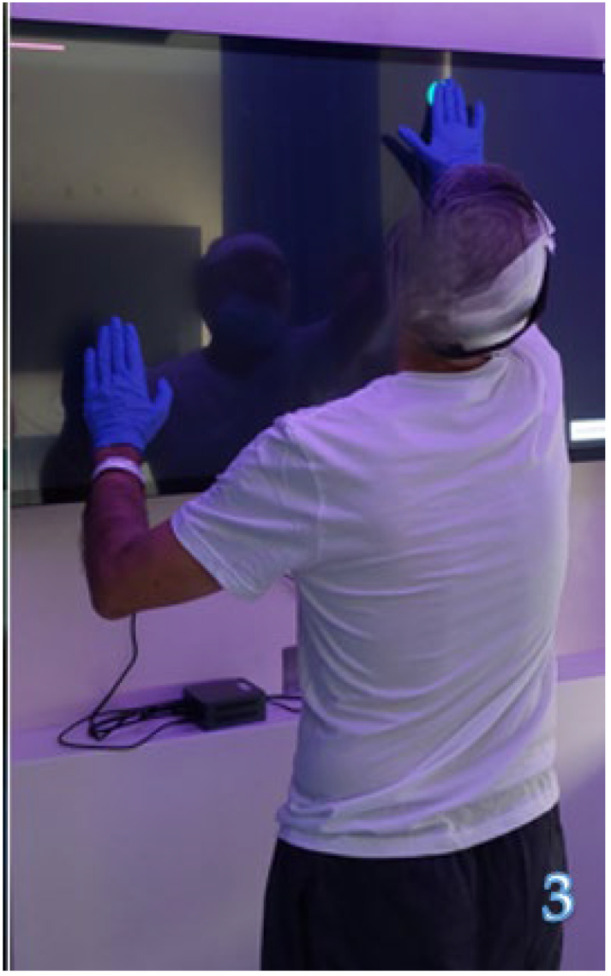
u-Touch.

**Figure 4:**
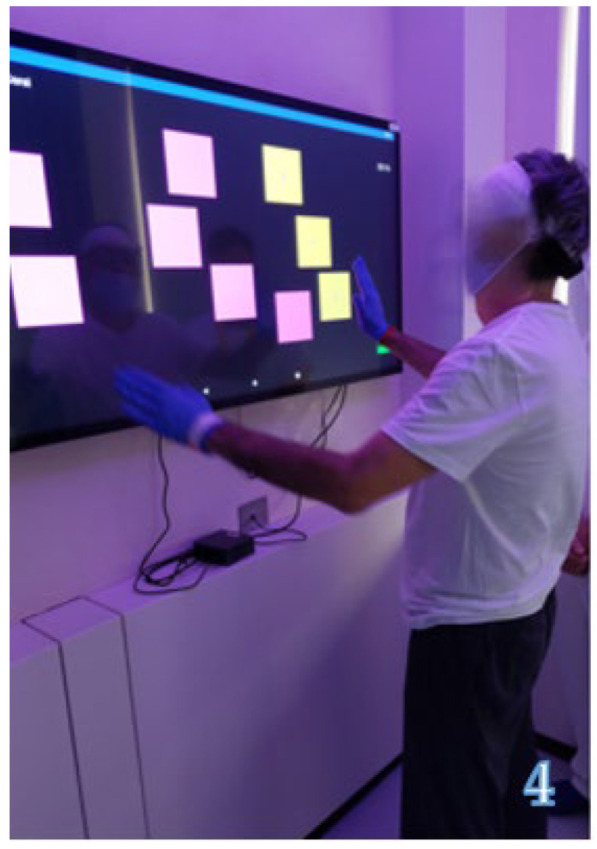
Digitalized Corsi test.

**Figure 5:**
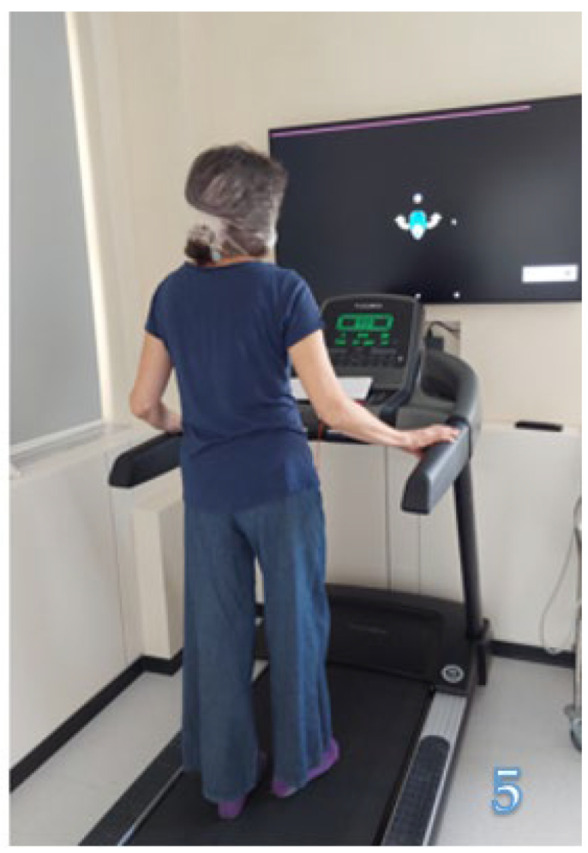
u-Read.

Alongside regular physical training at the gym, focused on improving posture and muscle strength, the patients underwent tailored rehabilitation, which involved the following exercises:

- v-Gym: two minutes of head rotations in the horizontal and vertical planes, progressively increasing the speed, during which the patient is asked to identify the numbers that appear on the touch screen arranged in front of the patient (Figure 2).
  Aim: vestibulo-ocular reflex training, minimizing dizziness, and improving visual stability.
- u-Touch: the patient has to touch spots which appear randomly on the touch screen (Figure 3).
  Aim: training coordination and improving performance in daily activities.
- Digitalized Corsi test: the patient is presented with a variable number of squares on the touchscreen which sequentially change color, forming a series that increases in number during the test; the patient is asked to memorize each series and to reproduce it on the touch screen (Figure 4).
  Aim: visuospatial working memory training.
- u-Read: the patient has to identify and memorize a series of randomly generated letters (single or double ones) while rotating their head on the horizontal or vertical plane at a progressively increasing speed and then write them down on a keyboard (Figure 5).
  Aim: improving visual stability, visual working memory, and the ability to shift between different activities.

The data collected were age, sex, timing of rehabilitation program, bedside examination, vHIT (in particular, HIMP and SHIMP of the lateral semi-circular canal of the healthy and pathological side), functional HIT (lateral semi-circular canal), and DHI. 

All the information was stored in a database (Software Excel for Microsoft 365).

Statistical analyses were performed with the IBM^®^ SPSS^®^ Statistics 20 software. For data comparisons, depending on variable distribution, a *t*-test, or a Wilcoxon test, were used to analyze the significance of measurement differences before and after the rehabilitation. Differences with a *p*-value below 0.05 were considered to be significant.

## 3. Results 

The data of 52 patients were analyzed. The mean age was 59 years old and 65% of patients were female (the main demographics are illustrated in Table 1). 

**Figure 6:**
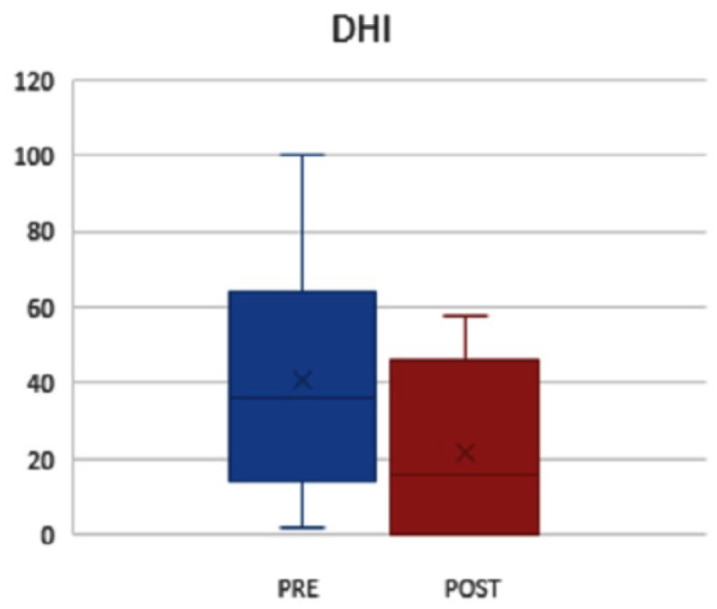
DHI prescores pre- (mean score 50.20, SD 23) and post-rehabilitation (mean score 50.20, SD 22); *p* = 0.01.

Figure 6 shows a comparison of the values in the DHI before and after the intervention. There was a significant difference in the values of the DHI before and after the rehabilitation program, as they were considerably lower in the post-treatment time.

The function of the horizontal semicircular canals at high frequencies of stimulations was assessed with vHIT from ICS Impulse (Otometrics/Natus, Denmark). 

Significant differences in Head Impulse Paradigm (HIMP) values were found among pathological and healthy sides before and after the treatment. 

**Figure 7:**
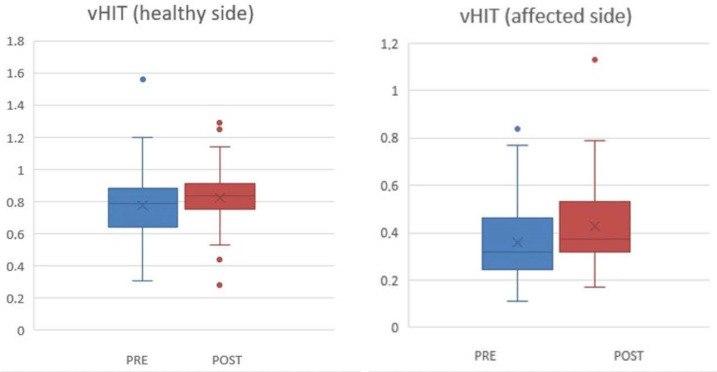
vHIT (HIMP) values before and after rehabilitation in the healthy and affected side, respectively. The box and whiskers illustrate the vHIT values in the healthy side pre- (mean value 0.77, SD 22) and post-rehabilitation (mean value 0.82, SD 0.19), *p* = 0.26, and in the pathological side pre- (mean value 0.36, SD 0.16) and post-rehabilitation (mean score 0.40, SD 0.18), *p* = 0.19. SD = Standard Deviation.

The VOR values on the damaged side remained unchanged; however, even on the healthy side, there were no significant modifications (*p* = 0.26) (Figure 7).

The Suppression Head Impulse test (SHIMP) revealed the same curve of HIMP, with improvement values after rehab (*p* 0.038). 

**Figure 8:**
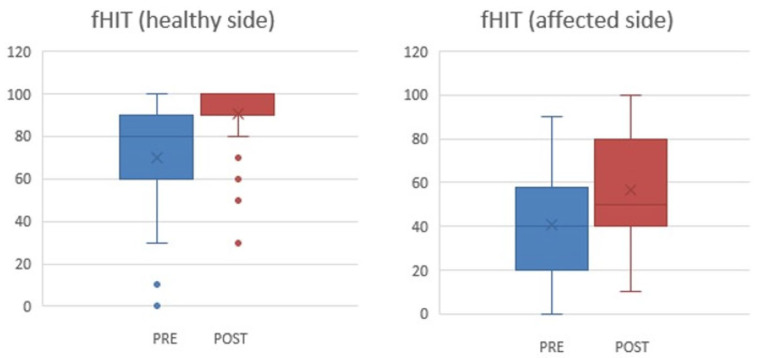
fHIT values before and after the rehabilitation program on the healthy and affected side, respectively. The box and whiskers illustrate the fHIT values for the healthy side pre- (mean value 70, SD 27) and post-rehabilitation (mean value 90.2, SD 16), *p* < 0.001, and for the affected side pre- (mean value 40, SD 25) and post-rehabilitation (mean value 57, SD 25), *p* 0.004.

The outcomes of fHIT (Figure 8), which explores the higher connection of the vestibular system with visual and cerebellar pathways, and thus central dynamics of the vestibular compensatory process, diverge from vHIT: both pathological and healthy side results improved substantially after training (*p* 0.004 and *p* < 0.001, respectively).

The BSE included an evaluation of nystagmus (Ny) with inhibition of fixation using infrared video goggles.

Spontaneous, positional, and evoked Ny revealed a trend in improvement after rehabilitation, despite no statistically significant values (*p* 0.065 and 0.063). 

The Romberg test before and after the intervention showed a significant improvement (*p* 0.004), and this was also reflected by the Unterberger test (*p* 0.022). 

## 4. Discussion

After surgical removal of VS, the vast majority of patients experience dizziness and unbalance. A spontaneous recovery process can take months to occur, and unfortunately, not all patients experience improvements in their symptoms.

The goal of this study was to evaluate the impact of tailored vestibular therapy in the postoperative time to expedite surgical recovery and minimize vestibular long-term effects. 

The guidance of experienced physiotherapists was crucial to supporting the patients, providing real-time feedback, and preventing injuries and erroneous exercise techniques.

The program comprised balance training, cognitive and executive function exercises, and visuospatial working memory tasks, which are essential in daily activities [14].

In our cohort of patients, the digitalized Corsi test was administered with the final goal of improving short- and long-term working memory [15,16].

The projection of the target on the retina during movement, named optic flow, is critical for direction, path integration, and orientation [17]. The role played by the visual system during navigation is prominent, thus the training of the different patterns of optic flow via visual training is essential to improve compensation. 

The restitution of self-motion integration (vestibular, proprioceptive, optic) and external sensorimotor information is a major goal of rehabilitation.

Our customized program is designed to train visual–spatial working memory alongside vestibular, proprioceptive, and visual systems, as this is crucial to improving quality of life. The vHIT values revealed the presence of a residual vestibula-ocular reflex function (VOR) higher than 0.6 in 11% of the casuistry, with unexpectedly high postoperative HIMP values, despite the TLA approach and VS excision. This finding was also observed by Černý et al. in 24% of their cohort of patients who underwent VS resection via the retro-sigmoid approach [18].

Even other researchers demonstrated that patients may still have vestibular function (determined by the vHIT) even after VS resection, underlining the ways in which the vHIT is not a useful tool for estimating overall vestibular performance [19].

Moreover, other authors observed the presence of residual VOR gain in cases of labyrinthectomy or neurectomy for Meniere disease [20,21].

Various theories have been developed around this topic, of which the hypothetical explanations were the presence of residual vestibular fibers, anastomosis between cochlear and inferior vestibular nerves, anatomical variants, incomplete deafferentation, or even recording artifacts.

However, in our cohort, in consideration of the TLA approach chosen to excise the tumor, residual vestibular function was not expected. It is established from research on humans and animals that even in cases of full vestibular deafferentation, the eye is able to react to high-acceleration stimuli, probably because of the head thrust’s excitation–inhibition effects on both vestibules [10]. Further studies in this field are required to better investigate these outcomes. 

Another interesting finding of our study was the gain improvement in the healthy vestibular system after rehabilitation, in contrast with the hypothesis of central compensation inhibition of the contralateral vestibular pathway [22].

The most important test for detecting VOR impairment is the video-HIT. The v-HIT provides gain value output that summarizes the behavior of VOR as the ratio of a measure of eye movement to the corresponding head movement. 

Whilst the vHIT analyses the VOR determines whether the eye movements are adequate for the head stimulus, on the other hand, the fHIT provides information on the VOR’s main purpose, which is the clear vision of the target and its cognitive integration.

Few authors observed the absence of a correlation between vHIT and fHIT outcomes, underlying how VOR gain may not correlate with the ability to read the target [23].

Interestingly, the scores of fHIT improved on both the pathological and healthy sides after the rehabilitation, revealing how intensive training improves the processing and integration of the vestibular stimuli, despite the low VOR values.

The mean fHIT measures before the rehabilitation were 40% on the pathological side and 70% on the contralateral and showed a considerable improvement after (57% and 90%, respectively). This is extremely important since the risk of falling appears to increase by 3.9 times when fHIT scores are below 80% [24].

The subjective assessment of vertigo using DHI showed an overall reduction in scores of 16.2 points after ten sessions of training, supporting the role of an intensive rehabilitation program. 

Moreover, our patients were encouraged to pursue training at home under telemedicine supervision by means of the Beon V Gym app for tablets. The improvements were therefore expected to continue over the following months. 

The main result of this study is the evidence that unlike vHIT, which only provides a raw assessment of VOR values and does not account for the functional effects of rehabilitation, fHIT allows us to measure the impact of rehabilitation on the overall compensation process, which is otherwise only subjectively estimated by DHI.

We hope that our research will help to disseminate the use of the fHIT, helping to deepen our understanding of the sophisticated neurological mechanisms and pathways involved in inputs and outputs directed to and from the vestibular nuclei.

### Drawbacks

The first limitation is the short-term follow-up of the patients included since the rehabilitation program is performed during the admission. Following their discharge, patients received follow-up care at home using the Beon V Gym tablet 1.1.5 app. Additional analysis of long-term vestibular outcomes will be a topic of future publications. 

Moreover, we decided to exclude patients affected by VS classified as large or giant (according to Kanzaki et al.) [25]. Tumors of this size may necessitate enlarged TLA combined with transapical extension and may be associated with cranial nerve impairments (such as VI or lower cranial nerves), or intracranial hypertension, which often requires pre-operative ventriculoperitoneal shunting. For these reasons, we preferred to select a cohort of homogeneous patients affected by VS < 3 cm. However, further studies focusing on large and giant VS are desirable. Finally, the current study was not designed to investigate comorbidities or the influences of sex and age on the outcomes. Further investigations with a larger sample size can help clarify possible differences among specific subgroups of patients.

## 5. Conclusions

To our knowledge, this is the first study to include fHIT evaluation in the assessment of patients after removal of VS.

The outcomes revealed that extensive postsurgical rehabilitation is an important aspect of the compensatory process and a crucial component of treatment, favoring vestibular and central compensation even just a few days after surgery.

## Figures and Tables

**Table 1:** Descriptive findings of the cohort.

	Mean Value
Age	59 years old (Range 36–83)
Sex	
Male	18 (35%)
Female	34 (65%)
Affected Side	
Right	22 (42%)
Left	30 (58%)

## Data Availability

The data presented in this study are available in this article.
